# Expert Consensus on Characteristics, Etiology, and Management of Chorioretinal Atrophy in Patients Treated with Voretigene Neparvovec

**DOI:** 10.1016/j.xops.2026.101090

**Published:** 2026-01-29

**Authors:** M Dominik Fischer, Isabelle Audo, David Gaucher, Frank G. Holz, Line Kessel, Stephen Russell, Katarina Stingl, David L. Rousso, Rainer Maier, Andreas Clemens, Aaron Nagiel, Bart P. Leroy

**Affiliations:** 1 Oxford Eye Hospital, University of Oxford NHS Foundation Trust and NIHR Oxford Biomedical Research Centre, Oxford, UK; 2 Nuffield Laboratory of Ophthalmology, Department of Clinical Neurosciences, University of Oxford, Oxford, UK; 3 University Eye Hospital, Centre for Ophthalmology, University Hospital Tübingen, Tübingen, Germany; 4 Sorbonne Université, INSERM, CNRS, Institut de la Vision, Paris, France; 5 Centre Hospitalier National d'Ophtalmologie des Quinze-Vingts, National Rare Disease Center REFERET and INSERM-DGOS CIC 1423, Paris, France; 6 Department of Ophthalmology, Nouvel Hospital Civil, Strasbourg University Hospital, FMTS, University of Strasbourg, Strasbourg, France; 7 Department of Ophthalmology, University of Bonn, Bonn, Germany; 8 Department of Ophthalmology, Copenhagen University Hospital, Rigshospitalet, Copenhagen, Denmark; 9 Department of Clinical Medicine, University of Copenhagen, Copenhagen, Denmark; 10 The University of Iowa Institute for Vision Research, University of Iowa, Iowa City, Iowa; 11 Center for Rare Eye Diseases, University of Tübingen, Tübingen, Germany; 12 Spark Therapeutics, Inc., Philadelphia, Pennsylvania; 13 Presently at Genentech, Inc., South San Francisco, California; 14 Novartis Pharma AG, Basel, Switzerland; 15 Roski Eye Institute, Department of Ophthalmology, Keck School of Medicine, University of Southern California, Los Angeles, California; 16 The Vision Center, Department of Surgery, Children's Hospital Los Angeles, Los Angeles, California; 17 Department of Ophthalmology & Centre for Medical Genetics, Ghent University Hospital & Ghent University, Ghent, Belgium; 18 Department of Head & Skin, Ghent University, Ghent, Belgium

**Keywords:** Chorioretinal atrophy, Gene therapy, Inherited retinal dystrophy, *RPE65*, Voretigene neparvovec

## Abstract

**Purpose:**

Voretigene neparvovec (VN), developed for the treatment of inherited retinal dystrophy (IRD) associated with confirmed biallelic *RPE65* mutations (*RPE65-*IRD), is the first approved retinal gene therapy. Recently, reports have emerged of chorioretinal atrophy (CRA) developing in a subset of patients treated with VN. Although researchers have started to investigate the etiology, detection, classification, and clinical course of CRA after treatment with VN, information gaps remain. Here, we review current data on CRA in patients treated with VN, propose standardized terminology for describing CRA, and provide guidance on the management of CRA in patients treated with VN.

**Design:**

Review of the scientific literature and expert consensus.

**Participants:**

An international group of experts in IRD with experience treating with VN.

**Methods:**

A literature search of PubMed was performed using broad search terms to return all publications relating to VN, which were manually screened for relevance to CRA. The expert panel discussed the literature and proposed updated consensus nomenclature and suggestions for monitoring.

**Results:**

According to most reports, development of CRA does not impact visual and functional outcomes after subretinal VN injection. Longer follow-up is needed to ascertain whether CRA continues to enlarge and whether treatment outcomes can be maintained. Chorioretinal atrophy growth might be caused by a combination of multiple factors, and several hypotheses on CRA etiology based on published clinical evidence are discussed. Standard terminology and metrics for CRA would support these future research efforts. We propose using the term “injection site CRA” to refer to CRA which develops specifically at the location where the cannula touches down onto the retina. For all other CRA, we propose defining atrophy based on its retinal localization, as either “central CRA” or “peripheral CRA.” Regular follow-up visits using a combination of imaging modalities with efficacy readouts are recommended to track efficacy and adverse outcomes, including CRA.

**Conclusions:**

Numerous questions remain regarding causes of CRA in *RPE65-*IRD treated with VN, which will require further clinical experience and research to answer. Here we propose terminology and key metrics for monitoring CRA to support such future efforts.

**Financial Disclosure(s):**

Proprietary or commercial disclosure may be found in the Footnotes and Disclosures at the end of this article.

Inherited retinal disorders (IRDs) encompass a diverse group of rare conditions caused by mutations in >300 different genes,^1^ of which some are progressive while others are not. The *RPE65* gene encodes a protein responsible for isomerohydrolase activity, converting all-*trans* retinyl ester to 11-*cis* retinol in the retinal pigment epithelium (RPE). Biallelic pathogenic variants in *RPE65* are linked to a spectrum ranging from Leber congenital amaurosis over early-onset retinal dystrophy to autosomal recessive retinitis pigmentosa, mostly with early onset in childhood,^2^ and phenotypes resembling congenital stationary night blindness, such as fundus albipunctatus have also been described.^3^ The single heterozygous variant p.(Asp477Gly) (c. 1430A>G) in *RPE65* has been associated with autosomal dominant retinitis pigmentosa with choroidal involvement.^4^ More recently, the p.(Glu519Lys) (c.1555G>A) in *RPE65* has also been associated with an autosomal dominant macular dystrophy.^5^

The prognosis for *RPE65*-related IRD (*RPE65*-IRD) is typically poor. Individuals with pathogenic variants in both *RPE65* gene copies often experience severe vision loss from an early age, with most patients eventually progressing to complete blindness.^6^ Early-stage symptoms include impaired dark adaptation, night blindness, reduced light sensitivity, peripheral vision loss, low or declining best-corrected visual acuity, and nystagmus.^7^^,^^8^ In early disease, the fundus may appear mostly normal or with a retina appearance with small, white spots, similar to those seen in fundus albipunctatus.^3^ More advanced disease is associated with outer retinal atrophy, including the foveal, parafoveal, and peripheral retina.^8^, ^9^, ^10^

*RPE65*-IRDs result in considerable burden to patients and their families and can have a devastating impact on quality of life. These conditions significantly impact patients in their daily activities and affect aspects of identity and autonomy, with studies linking visual impairment to reduced social engagement, self-confidence, and vision-related quality of life, as well as to increased rates of depression.^11^

Historically, treatment options for individuals with biallelic *RPE65* mutations were limited to supportive care, including optimal refractive correction and low-vision aids.^10^ Voretigene neparvovec (VN) is a gene therapy for IRD associated with confirmed biallelic *RPE65* mutations, which received US Food and Drug Administration approval in December 2017 and European Medicines Agency approval in November 2018. Voretigene neparvovec was the first and still remains the only gene therapy approved to treat an inherited retinal disease. It is indicated for children and adults with the clinical diagnosis of an inherited retinal dystrophy caused by confirmed biallelic *RPE65* mutations and who have sufficient viable retinal cells.^12^

When injected into the subretinal space, VN employs an adeno-associated viral vector serotype 2 (AAV2) capsid as a delivery vehicle to transduce RPE cells with a cDNA encoding normal human RPE65 protein.^12^ This functional RPE65 is intended to restore visual function and improve vision in patients with adequate viable retinal cells.^6^

Voretigene neparvovec received regulatory approval based on findings from 2 phase 1 trials (study 101 and 102 follow-on study), and the first randomized, controlled phase 3 gene therapy trial for an inherited eye condition.^6^^,^^13^ In phase 3, improvements were observed as early as 30 days posttreatment,^6^ with results sustained at 4 years.^14^

When VN initially received approval, its safety profile was described as comparable to that of vitrectomy, with the additional potential for vector-related inflammation.^6^^,^^13^ However, in the years after approval, reports emerged on the occurrence of progressive thinning and degeneration of the choroid and retina, known as chorioretinal atrophy (CRA), developing in a subset of patients treated with VN.^15^ In response, a retrospective review of fundus photographs from 39 of 41 patients in the VN clinical development program was performed, which revealed increased occurrence of CRA in treated eyes over time,^12^ although the study was limited and did not allow CRA observed after VN treatment to be distinguished from injection site CRA or from CRA observed as part of natural disease progression, limiting clinical interpretation.

Researchers have shown an increased interest in CRA and started to address the etiology, classification, and clinical course of CRA after treatment with VN. However, the gain in knowledge and our understanding of the causes of VN-related CRA remain limited, including a generally agreed-upon description and definition for CRA observed in the context of VN treatment, as well as identifying potential risk factors. Since VN is the first and only approved retinal gene therapy, there is also little evidence available to help determine whether CRA is specific to treatment with VN or could be relevant to other ocular gene therapies in the future.

The objective of this publication is to review current evidence on CRA in patients treated with VN, propose a standardized terminology for describing CRA so as to provide a framework for future research, and provide guidance on the management of CRA in patients treated with VN, according to current knowledge and expert recommendations.

## Methods

A literature search of the PubMed database was performed using broad search terms to return all publications relating to VN. These were then manually screened for relevance to CRA, by title and abstract or as full text. Non-English language publications and nonhuman studies were excluded.

An international group of experts in IRD with experience treating patients with VN surveyed and discussed the published literature reporting cases of CRA in VN-treated IRD to gain insights into current definitions and classifications and proposed an updated consensus nomenclature with suggestions for monitoring.

## Results

After the review of the records returned by the database search, 19 reports were retrieved from the published scientific literature on atrophy after treatment with VN. Details of these are summarized in Table 1. The first reported description of CRA in a subset of patients after subretinal VN was made in 2022 by Gange et al.^15^ They presented a series of patients who developed perifoveal CRA, with 8 out of 10 patients experiencing it bilaterally. Chorioretinal atrophy appeared both within and outside the subretinal bleb area in 10 (55.5%) eyes, solely within the bleb area in 7 (38.9%) eyes, and exclusively outside the bleb area in 1 (5.5%) eye. The researchers determined the subretinal bleb area based on the extent and location of the bleb on intraoperative fundus photos, but it should be noted that this definition does not account for potential expansion or movement of the bleb between the end of surgery and its postadministration absorption. Additionally, 13 eyes with reliable preoperative and postoperative visual fields demonstrated improvement after surgery, of which 3 (23.1%) showed paracentral scotomas related to the CRA. Despite these changes, visual acuity improved or remained stable in 15/18 (83%) eyes, likely due to the fovea being spared.^15^

**Table 1:** Publications of CRA Associated with VN Treatment

Publication	Study Type	Study Size	Follow-Up Duration	Chorioretinal Atrophy					
				Incidence	Time to Detection of CRA	Imaging Modality Used to Monitor CRA	Definition/Description	Observations	Notes on Surgical Technique
Gange 2022^15^	Multicenter retrospective chart review	18 eyes of 10 patients	Mean, 11.3 months	N/A	Mean, 4.7 months	UWF pseudocolor FP	Patients were identified as having perifoveal CRA if: (1) the areas of atrophy were not directly related to the touchdown site of the subretinal cannula and (2) the area of atrophy progressively enlarged over time.	CRA progressively enlarged in all cases over follow-up. CRA developed within and outside the area of the subretinal bleb in 55.5% eyes, exclusively within the area of the bleb in 38.9% eyes, and exclusively outside the bleb in 5.5% eyes	Foot-pedal control used with maximum injection pressure of 16 pounds per square inch (PSI)
Giansanti 2022^23^	Case report	2 eyes of 1 patient	6 months	N/A	3 months (injection site), 6 months (elsewhere)	Color FP	Atrophy of the RPE (photoreceptors were already lost from areas of atrophy before treatment)	RPE atrophy at injection site, several perifoveal sites, and at the boundaries of or inside the vascular arcades, which progressively enlarged over follow-up	NR
Kessel 2022^16^	Retrospective chart review	23 eyes of 12 patients	6–15 months	26% (6/23)	<6 months	Color FP	NR	Subtle atrophy of the RPE and outer retina seen on OCT scans. New atrophic areas in 6 eyes, at the site of injection (2 eyes) or in areas were associated with prior vitritis and outer retinal infiltrates (4 eyes)	Foot-pedal assisted control used without formation of a pre-bleb
Kolesnikova 2022^21^	Case report	2 eyes of 1 patient	8 years	N/A	8 years (CRA may have developed earlier, but no follow-up occurred between the 2-year and 8-year visits)	Color FP, quantitative FAF	Definition by Gange et al used^15^	At 2 years, salt-and-pepper mottling and lacunae were seen superotemporally in both eyes. At 8 years, extensive bilateral superotemporal confluent CRA lesions were present	NR
Sengillo 2022^24^	Retrospective chart review	77 eyes of 41 patients	Up to 12 months	9% (7/77)	1 week in the patient with atrophy related to subretinal air	Color FP, FAF	NR	One eye developed extensive RPE atrophy with IS/OS junction loss, thought to be related to the use of subretinal air. Three cases with high myopia and related bilateral baseline RPE atrophy progressed more rapidly after VN therapy, with atrophy extending beyond the area of the bleb. All 7 eyes were included in the Gange et al case series	Implemented modifications described by Gange et al^15^
Testa 2022^25^	Retrospective chart review	12 eyes of 6 patients	6 months	50% (6/12)	6 months	SD OCT	Atrophy exceeding the retinotomy site	Focal retinal atrophy observed in retinal midperiphery (beyond vascular arcades) identified by evident loss of the RPE (i.e., a variable degree of translucency with a detectable choroidal vasculature)	According to protocol recommended in phase 3 study^6^
Kiraly 2023^22^	Retrospective study	12 eyes of 6 patients	Mean, 8.2 months	100% (12/12) atrophy at injection site; 83% (10/12) atrophy away from injection site	As early as 1 week in one patient	UWF pseudocolor FP	Retinal atrophy, defined as the development of new atrophy post gene therapy	10/12 eyes developed some retinal atrophy (8 mild, 2 severe) away from areas of injection. One case had retinal atrophy involving the fovea	Foot-pedal control used with maximum injection pressure of 10 PSI over 30–60 s
Reichel 2023^19^	Retrospective chart review	13 eyes of 8 patients	Mean, 15.3 months	100% (13/13)	Changes in FAF preceded the development of atrophy, evident 2 weeks after surgery	UWF FP and FAF	Postoperative retinal atrophy was defined as new areas of retinal atrophy exceeding the retinotomy site. The areas of retinal atrophy were further divided into areas within and outside the bleb (as per documented bleb location at end of surgery).	Atrophy beyond the retinotomy site was seen in all eyes. Areas of retinal atrophy developed within the bleb area in all 8 patients and outside the bleb in 3 patients. In most patients, atrophy increased with time over year 1.	Foot-pedal control used with maximum injection pressure of 10 PSI over 30–60 s. No subretinal pre-bleb. In some patients, 2 or 3 retinotomies were used.
Stingl 2023^17^	Retrospective cohort study with longitudinal follow-up	71 eyes of 38 patients	Up to 48 months	28% (20/71)	NR	Color FP and FAF	Post-VN perifoveal CRA was defined as growth around the arcades and mid-periphery beyond the retinotomy site.	Decreased autofluorescence seen on FAF and decreased pigmentation on fundus photography, as well as thinning or loss of the outer retina and RPE on OCT. CRA was more frequent in patients with better initial postoperative FST improvement.	Foot-pedal system used
Bommakanti 2024^26^	Multicenter retrospective analysis	27 eyes of 14 patients	Mean, 2.2 years	14% (27/187 total treated eyes)	NR	UWF SLO or color FP	Atrophy was defined as regions which satisfied ≥2 of the following criteria: (1) partial or complete depigmentation of the retinal pigment epithelium; (2) round shape; (3) sharp margins; and (4) increased visibility of underlying choroidal vessels. Atrophy was classified based on the most prominent feature into 3 types: atrophy at the injection site (touchdown); nummular areas of atrophy predominantly involving the periphery (nummular); and atrophy predominantly in the perifoveal region (perifoveal).	15 eyes had >1 type of atrophy. Bilateral atrophy occurred in 13 patients, and the same subtype of atrophy tended to occur in both eyes. Perifoveal atrophy grew the most rapidly, while touchdown atrophy grew the least rapidly.	Foot-pedal control was used with a maximum injection pressure of 16 PSI, no pre-bleb
Dormegny 2024^27^	Retrospective case series	6 eyes of 3 patients	18–24 months	0% (0/4) in ILM peel eyes 100% (2/2) in non-ILM peel eyes	3 months	Fundus biomicroscopy, NMR, UWF, and SD-OCT	NR	Multiple focal macular atrophic areas developed along the arcades, one of them including the injection site. Atrophy progressed during follow-up with progressive confluence. Preinjection ILM peeling preserved eyes from injection site CRA but not from other types of CRA	Focal ILM peeling was performed at the site of injection in 4 eyes
Fischer 2024^28^	Prospective, registry-based observational study	183 eyes of 103 patients	Mean, 0.8 years	10% (19/183)	Mean: 27.5 days for injection site atrophy and 101.5 days for progressive atrophic changes	NR	CRA was defined as a grouping of the following TEAEs: retinal degeneration, retinal depigmentation, and atrophy at the injection site	18 eyes developed atrophy at the injection site and 8 eyes had retinal degeneration reported as “progressive atrophic changes in the retina.” All CRA events reported were of mild severity.	Automated injection system used in 23.5% of eyes. More eyes with CRAs were treated using the automated injection system than eyes without CRAs (52.5% vs. 20.1%).
Ku 2024^29^	Retrospective chart review	4 eyes of 4 patients	5–34 months	75% (3/4)	5–22 months	Widefield FP	NR	Unilateral perimacular atrophy observed after VN treatment but was not observed in the fellow eyes which had been treated with a different viral vector 6-10 years earlier as part of a phase I/2 trial (NCT00749957).	Saline pre-bleb
Lorenz 2024^30^	Retrospective case series	30 eyes of 19 patients	Median, 15.1 months	50% (13/26)	12 months	UWF FP, infrared reflectance, blue-light FAF	NR	Central and/or peripheral CRA outside the large vascular arcades developed or enlarged. New CRA was also observed at the site of retinotomy in 11 of the 13 eyes that developed new or accentuated central and/or peripheral CRA.	No foot pedal, no pre-bleb
Melillo 2024^31^	Retrospective cohort study with longitudinal follow-up	24 eyes of 12 patients	At least 12 months	33% (8/24)	6 months	Color FP	Newly emerged areas of retinal atrophy with evident loss of RPE (i.e., a variable degree of translucency with a detectable choroidal vasculature) exceeding the retinotomy site	4 patients developed multifocal retinal atrophy in both eyes at 6 months. Fundus imaging at 12 months showed expansion of atrophic lesions. In all cases, these lesions were located within and around the subretinal bleb area.	According to protocol recommended in phase 3 study.^6^ In some patients, 2 or 3 retinotomies used.
Stingl 2024^18^	Retrospective analysis	11 eyes of 6 patients (some previously included in Stingl et al)	24 months	N/A	3 months	FAF	NR	CRA expanded in all eyes over 24 months after surgery. The areas of greatest gain in the number of functionally rescued rods were prone to be the initial spots of atrophy growth.	NR
Daruich 2025^32^	Retrospective chart review	12 eyes of 6 patients	12 months	Injection site atrophy in 91.6% (11/12) and perifoveal atrophy in 8.3% (1/12)	Mean, 4.1 months	UWF FP	NR	The mean diameter of CRA increased from 1.3 mm at diagnosis to 1.6 mm at 12 months. CRA outside the injection site but within the area of the bleb was detected in one eye.	According to protocol recommended in phase 3 study.^6^

CRA = chorioretinal atrophy; FAF = fundus autofluorescence; FP = fundus photography; ILM = internal limiting membrane; IS/OS = inner segment/outer segment; N/A = not applicable since patients were selected on the basis of having CRA; NMR = nonmydriatic retinography; NR = not reported; RPE = retinal pigment epithelium; SD-OCT = spectral-domain OCT; SLO = scanning laser ophthalmoscopy; TEAE = treatment-emergent adverse event; UWF = ultra-widefield; VN = voretigene neparvovec.

In a series of 23 eyes, Kessel et al observed new atrophic areas in 6 eyes (26.1%) and intraocular inflammation, specifically vitritis, in 9 eyes (39.1%). In one case, atrophy development occurred specifically in an area where outer retinal infiltrates had been detected on OCT scan. The researchers considered these outer retinal changes to be related to an inflammatory event since they appeared with the vitritis and resolved on immunosuppressant therapy, suggesting a link between CRA and inflammation.^16^ Stingl et al noted that CRA growth was found to follow a similar pattern in the majority of cases, beginning with round lesions visible on fundus photography and fundus autofluorescence (FAF) which over time progress and form confluent lesions.^17^ The investigators observed a correlation of the development of CRA with the efficacy of the treatment, with a greater improvement in full-field stimulus threshold (FST) outcomes in patients who developed CRA. In another publication from the same group, the authors note that the first clear sign of CRA is usually present by the third month after treatment, and that from this point the CRA enlarges rapidly until month 9 or 12, after which expansion usually slows.^18^ This publication also demonstrated that retinal areas which show a high number of reactivated rods, but which experience suboptimal functional rescue in light sensitivity at month 1 (before the development of any CRA), are the areas prone to be where the CRA starts to grow a few weeks later. In contrast, retinal areas showing the greatest functional response were consistently spared from degeneration.

Reichel et al recommended the use of FAF as a tool for early monitoring of CRA, noting that marked areas of decreased autofluorescence were visible as early as 2 weeks after surgery for most patients. Retinotomy-associated CRA was typically observed at the injection site. Hypoautofluorescent areas on FAF correlated initially with disruption of photoreceptor outer segment layers and occurrence of reflective material, followed by progressive outer retinal degeneration. Although the RPE cell layer was challenging to differentiate on OCT, the hypertransmission defects and decreased autofluorescence on FAF indicating RPE involvement were obvious.^19^ Short-wavelength FAF imaging can be challenging in patients with *RPE65*-associated IRD due to the relatively low FAF signal and poor fixation. However, the ability to record a reproducible FAF image often improves after VN treatment,^20^ and FAF images can be recorded in most cases for longitudinal follow-up (after treatment) to detect changes in the FAF signal. A loss would typically indicate RPE atrophy. Interestingly, Kolesnikova et al^21^ have also demonstrated an increase in macular autofluorescence 6 and 8 years after VN treatment in a patient with CRA affecting the peripheral retina.

In a UK-based retrospective study, one of 6 patients who developed retinal atrophy had foveal involvement, where the atrophy developed gradually and extensively outside the treated area. This patient had large improvements in FST of approximately –34 dB in both eyes, despite the presence of atrophy.^22^ This pattern of good functional outcomes despite the presence of CRA was noted in the majority of published reports.

### Possible Causes of CRA in VN-Treated IRD

The cause of CRA that has been observed after VN treatment is believed to be multifactorial, reflecting the limited knowledge and lack of a clear understanding of CRA at present. Several hypotheses have been put forward to explain the etiology of CRA after treatment with VN in patients with IRD. These include surgical factors, metabolic stress, vector toxicity, an immune or inflammatory response, phototoxicity, and natural disease progression (to which patient-related factors such as age and degree of degeneration may contribute).

#### Surgical Factors

The surgical delivery procedure itself is one factor that could potentially contribute to the development of CRA at the injection site and surrounding bleb area. Voretigene neparvovec delivery involves the induction of a temporary focal retinal detachment using a fluid jet to form a subretinal bleb.^19^ Mechanical trauma resulting from this process could cause transient or permanent damage to the outer retina, including loss of photoreceptor outer segments or RPE cells.^27^ Although the retina ultimately reattaches quite quickly after the procedure, it is thought that recovery of the retinal tissues might be impaired in IRD patients, potentially leading to CRA development.^19^

Higher injection rates, with resulting higher speed of the fluid jet, may be associated with more damage and increased risk of CRA, particularly within the bleb region.^33^ This is of particular concern in young patients and those with end-stage disease and intraretinal pigment migration, in whom higher pressures are sometimes required to induce a retinal detachment due to a strong adherence between the neuroretina and the RPE.^15^^,^^20^^,^^33^ In the authors’ experience, higher pressures are also required in those patients who have end-stage disease due to the fact that there is cicatricial damage due to intraretinal pigment migration. In response, some investigators have recommended using a foot-pedal system to administer the injection at a controlled, stable pressure.^15^^,^^20^ Foot-pedal control was not used in the phase 3 study of VN, in which there were no reports of CRA,^6^ and results from the PERCEIVE registry study suggested that the use of an automated injection system was more common in patients who subsequently developed CRA than those who did not.^28^ The underlying reasons for this counterintuitive observation remain unclear.

Several other variations on the surgical procedure have been described in the literature, with the aim of reducing the risk of CRA development. These include the use of a saline prebleb, which it has been hypothesized may reduce the risk of vector toxicity;^15^ using multiple injection sites to create more than one bleb and thus reduce the mechanical stress on the retinal tissues;^31^^,^^34^ and performing an internal limiting membrane peel before injection, intended to reduce the required injection pressure.^15^^,^^27^ However, to date there is little evidence to suggest reductions in the incidence of CRA with any of these measures, and they may be associated with increased risk of complications of their own.

Ultimately, a number of observations, including variability in CRA development between identically treated patients, the bilateral nature of CRA in many patients, and development of CRA beyond the injection site, all suggest the involvement of other mechanisms in CRA development.^15^^,^^17^

#### Metabolic Stress and Functional Rescue

A sudden increase in the metabolic activity of photoreceptors and RPE cells resulting from the restoration of visual function has been proposed as a cause of CRA in the treated area outside of the injection site.^17^^,^^18^^,^^29^

It has been suggested that a degenerated retina, existing in a low metabolic state with reduced oxygen requirements and supply, may struggle to accommodate the renewed activity after treatment with VN. This hypothesis is supported by studies finding that initial improvement in FST correlates with higher risk of CRA development, suggesting that areas with significant local functional rescue experience high levels of metabolic activity that can in some cases lead to cell death.^17^^,^^18^ For example, a study which performed a retinotopic examination of improvements in rod function occurring before CRA expansion reported a spatial connection between local rescue effects 1 month after treatment and CRA at month 3. Results showed that, in a cohort of 11 eyes, CRA often started to develop in areas where a high number of rods with suboptimal functionality were reactivated by gene therapy. Areas where rods were able to gain high sensitivity were less likely to develop CRA, remaining functionally stable over 2 years.^18^ One interpretation of these results is that reactivation of a substantial number of rods lacking full functionality created an environment of metabolic stress. This may explain cell death in some areas but long-term preserved function in other areas. Interestingly, in a mouse model of retinal degeneration after gene therapy, the authors showed using untargeted proteomics that *PDE6B* gene restoration is followed by inactivation of proinflammatory proteins along with a strong increase in metabolic demand.^35^

However, an association between increased visual function and CRA development has not been observed by all researchers, with another group failing to find a positive correlation between FST and increased CRA,^30^ although this analysis included some patients with CRA at the injection site only, which may have influenced the results.

It is notable that the parafoveal CRA observed afrer treatment with VN shares certain anatomical features with the autosomal dominant *RPE65* phenotypes.^4^^,^^5^^,^^36^ The underlying mechanisms of autosomal dominant *RPE65* dystrophy remain poorly understood but may be linked to RPE65 activity, which would provide support for the metabolic stress/functional rescue hypothesis. Further investigation into autosomal dominant *RPE65* dystrophy could also provide insight into the specific pathways and processes involved in CRA development after VN treatment.

#### Vector-Related Toxicity

Some researchers believe that direct toxicity of the AAV2 vector to the photoreceptors and RPE may play a role in the development of CRA after treatment with VN.^15^ Understanding of the cause of potential vector-related toxicity is still incomplete, with suggestions including the effect of the AAV vector type and its effectiveness in transducing cells, the promoters used to drive *RPE65* expression, and immune responses due to prior AAV exposure, among others. In a preclinical study investigating the ocular toxicity of different AAV vectors, dose-dependent RPE loss and outer nuclear layer thinning were observed with vectors utilizing RPE-specific and broadly active promoters, including the chicken β-actin promoter used in VN (although the VN vector itself was not tested).^37^ Although difficult to test, the relative stoichiometric differences between *RPE65*-IRD patients may play a role. With 1.5 x 10^11^ vector genomes delivered in a 300 μL volume, the effective vector-to-cell ratio in the treated area can vary substantially between patients, depending on the degree of RPE loss,^38^ assuming that nonviable cells have been cleared and do not compete with viable RPE for vector binding.

It has been speculated that the use of a saline prebleb, rather than injection of the vector directly into the subretinal space, could moderate toxicity by reducing the concentration of the vector,^15^ but the effects of this on the effectiveness of treatment and risk of CRA are not yet well understood,^34^ and the practice is not part of the administration instructions included in the VN summary of product characteristics.^12^

#### Immune and Inflammatory Responses

Vector-related inflammation is another potential explanation for CRA after VN. The manufacturers of VN recommend a preoperative and postoperative immunomodulatory regimen of prednisone or an equivalent agent for 3 days prior to VN administration and 2 weeks after the procedure, including tapering after the first 4 days.^12^ Under this regimen, postoperative intraocular inflammation is generally mild with reduced signs of inflammation. Transient, low-grade ocular inflammation was observed in 2 of the 29 patients (6.9%) treated in the phase 3 trial of VN.^14^

There have been a small number of reports of postoperative ocular inflammation occurring in eyes that later developed CRA. A Danish group reported signs of intraocular inflammation, primarily vitritis, occurring after completion of immunosuppressant therapy in 9 of 23 eyes (39.1%) receiving VN.^16^ When both eyes were treated with VN within a single, extended period, inflammation was more frequent and severe in second-treated eyes, which received a shorter postoperative course of immunosuppressants. In one patient, outer retinal infiltrates appeared at the same time as the vitritis and were the site of later CRA.^16^

In the case series of patients with CRA after VN reported by Gange et al, bilateral ocular inflammation was observed in one patient,^15^ while in the PERCEIVE registry-based study, intraocular inflammation and/or infection related to the procedure were reported in 7 of 103 patients (6.8%), making it less common than CRA at the injection site or elsewhere, which was reported in 19 eyes of 13 patients (10.4% of eyes).^28^ Other researchers documenting cases of CRA after VN did not observe any signs of inflammation in their patients.^17^^,^^19^

However, inflammation associated with the anterior chamber is often limited to the immediate postoperative phase and may be less relevant to the long-term development of CRA than a chronic chorioretinal immune or inflammatory response. Hyperreflective foci resembling punctate inner choroidopathy have been observed to appear after VN treatment, in some cases at sites where CRA later developed.^30^ It has been suggested that subclinical immune responses may have an effect on the retinal cells, even when there is no obvious inflammation present.^19^^,^^29^ While investigators using adaptive optics ophthalmoscopy to follow 16 eyes treated with VN over the course of 12 months reported no signs of subclinical inflammation such as perivascular infiltrates or large cell migration,^39^ this does not exclude the potential presence of chronic inflammation at the chorioretinal level. However, it seems unlikely that postoperative inflammation is the sole cause of post-VN CRA. Indeed, subclinical immune reaction in retinal tissue is a known accompanying effect of cell death, part of the natural course of IRDs. Immune reactions in the retina in the areas of CRA may be a sequela of developing atrophy.

#### Light Sensitivity and Phototoxicity

Animal data suggest that excessive light may accelerate retinal dystrophies including Stargardt disease and *RPE65*-associated retinal diseases.^40^, ^41^, ^42^, ^43^ Another possible explanation for VN-related CRA is that the restoration or enhancement of retinal function increases its sensitivity to light and thus increases its susceptibility to light-induced damage.^17^

In rodent models, some *RPE65* variants result in slowed rhodopsin regeneration. Since light damage to photoreceptors is initiated by the excess absorption of photons by rhodopsin, lower levels of rhodopsin could potentially protect photoreceptors from light-induced damage.^41^ By restoring rhodopsin regeneration, treatment with VN could theoretically make the retina more vulnerable to light-related cell stress and ultimately to CRA.^17^

#### Natural Disease Progression

Finally, there is the possibility that the CRA observed in VN-treated patients is occurring as part of the natural history of *RPE65*-associated IRD, since progressive atrophy is a feature of the condition and areas of retina not exposed to VN remain untreated.

Arguing against this theory is the observation that the timing of CRA often correlates with treatment, and the rate of CRA growth exceeds that observed in historical cohorts of untreated patients, suggesting treatment-related origins.^15^^,^^20^ In the retrospective analyses performed by the groups of Reichel^19^ and Lorenz,^30^ CRA was not observed in untreated contralateral eyes, which again reduces the likelihood that natural history is the sole cause of this phenomenon. However, during natural history studies, peripheral degenerative changes may not always have been observed due to limited capabilities of adequately capturing the retinal periphery with previous generations of fundus cameras. In addition, the peripheral retina may also not have been the main area of interest of all authors. It is equally possible that peripheral retinal degenerative changes due to natural evolution of disease accelerate consequent upon additional inflammation after the surgical trauma of the vitrectomy as part of VN treatment.

#### Possible Risk Factors for the Development of CRA

Several potential risk factors for the development of CRA have been described by researchers, including age, degree of functional recovery, and the presence of myopia. Patients between school age and young adulthood seem to be more prone to developing CRA, with very young and older patients showing lower risk,^17^, ^18^, ^19^^,^^30^ although this observation should be considered in the context of the age distribution of the treated population. An explanation put forward to explain this pattern of CRA development is the metabolic theory for CRA development discussed above.^18^ According to researchers, preschool children and those with relatively well-preserved retinas may be less likely to develop CRA despite good functional rescue from VN treatment, because for these patients the functional reactivation of retinal cells does not represent a deleterious burden in terms of metabolic burden and oxygen consumption.^18^ Meanwhile, advanced disease with few remaining target cells (such as that often seen in older patients) is associated with minimal functional benefit from VN treatment, but also a relatively low risk of CRA development, possibly because much of the retina is already atrophic and the retinal metabolism changes only slightly after treatment. However, in younger patients, a robust increase in retinal sensitivity is often observed, which is thought to result in increased metabolic activity in RPE cells and rod photoreceptors. The metabolic theory suggests that, due to a certain stage of retinal degeneration, this increased metabolic demand represents an imbalance in the tissue which can trigger further cell deaths.^18^

Another hypothesis suggests that younger patients may exhibit a more pronounced immune response, which could trigger inflammatory reactions after gene therapy surgery, contributing to more pronounced CRA development in this group. Preschool-aged patients have been reported to show heightened immune responses after gene therapy without developing CRA,^44^ which appears to contradict this theory, but these findings are based on a small sample size which limits their generalizability. Further research is required to clarify the role of immune mechanisms in CRA development across different age groups.

The presence of myopia has also been linked to a higher risk of CRA in some studies. In the case series from Gange et al,^15^ 9 of the 10 patients who developed CRA after VN treatment were myopic, with a mean refractive error of –6.1 D. It was suggested that thinning of the choriocapillaris in highly myopic individuals could make them more susceptible to vector toxicity or inflammation.^15^^,^^24^ This might be a risk factor in individual cases.

## Discussion

Since the first report of CRA after treatment with VN by Gange et al^15^ in 2022, cases of CRA after treatment with VN are increasingly being documented and shared in the literature. The reported incidence of CRA occurring beyond the retinotomy site in patients post-VN treatment varies greatly, from 9% in some reports up to 100% in others. It is notable that, in most reports, development of CRA did not affect the expected visual and functional outcomes after subretinal VN injection. However, longer follow-up will be required to ascertain whether CRA continues to develop over time and, if this is the case, whether functional gains after treatment can be maintained.

Several hypotheses on the etiology of CRA in VN-treated IRD have been proposed, and combinations of multiple mechanisms may be involved, resulting in a spectrum of atrophic changes.^26^ As the first available subretinal gene therapy, there is limited opportunity to compare the safety profile of VN with other similar agents. However, as more such subretinal gene therapies become available, their adverse event profiles may provide further insights into the etiology of CRA. Numerous other gene therapies delivered via subretinal injection are currently under investigation for the treatment of a range of retinal diseases, some of which have reported ocular complications relevant to this review. Chorioretinal atrophies of the typical patchy appearance and rapid growth such as after VN have not been reported in any other gene therapy trial so far. However, RPE atrophic changes, including atrophy at the injection site, have been reported after these other therapies.

Subretinal timrepigene emparvovec, an AAV2 vector-based gene therapy for choroideremia, has shown adverse effects such as ocular inflammation and retinal detachment in some treated eyes with thin, degenerate tissues in a phase 3 study.^45^ In a phase I/IIa study, treatment with SAR422459, which utilizes an equine infectious anemia virus vector to deliver gene therapy for Stargardt disease, led to RPE atrophy at the retinotomy site in most of the participants, as well as increased hypoautofluorescent lesions in 6 patients and RPE thinning in 1 patient, albeit with no well-defined CRA such as that seen after VN injection.^46^

Several therapies utilizing an AAV8 vector for delivery are currently in development. Subretinal RGX-314 is delivered via an AAV8 vector for treatment of nonhereditary retinal diseases such as neovascular age-related macular degeneration and diabetic retinopathy. In a phase I/II study, RGX-314 treatment resulted in dose-related pigmentary changes in the inferior retinal periphery of some patients,^47^ which may reflect the development of retinal atrophy with pigment migration. These pigmentary changes were mostly asymptomatic; however, in 3 participants the changes occurred in the macula and were associated with retinal thinning and reduced visual acuity.^47^

In a phase I/II dose-escalation trial for retinal dystrophy due to biallelic *RLBP1* mutations, subretinal AAV8-*RLBP1* gene therapy caused dose-dependent intraocular inflammation, RPE atrophy, retinal atrophy at the retinotomy site, and subretinal pigmented deposits with secondary retinal atrophy near the retinotomy in higher dose cohorts.^48^ Meanwhile, cotoretigene toliparvovec (AAV8-*RPGR*) for X-linked retinitis pigmentosa was associated with steroid-responsive subretinal inflammation and hyperreflective deposits in patients receiving higher doses in a phase I/II study.^49^

A retrospective analysis of preclinical evidence in nonhuman primates treated with a rAAV2/8-based gene therapy for *PDE6A*-associated retinitis pigmentosa has reported dose-dependent, progressive retinal atrophy at the injection site. Six of 11 eyes (54.5%) injected with a high dose of vector and 3 of 11 eyes (27.3%) injected with a low dose of vector developed injection site atrophy, compared with none of the 17 eyes that received sham injection.^50^ In addition, CRA lesions in low-dose eyes tended to be smaller and progress more slowly than those in high-dose eyes. Results from a trial of *PDE6A* gene therapy in humans are expected soon and awaited with interest. In a recent study reporting on 4 children with *AIPL1*-LCA4 aged 1.0 to 2.8 years old at the time of treatment with rAAV8.hRKp.*AIPL1*, 1 child showed CRA at the injection site.^51^

Further research is needed to definitively confirm the complete etiology of CRA in VN-treated IRD, elucidate the baseline characteristics that could predict patients at higher risk of CRA, and confirm the best way to manage patients with IRD treated with VN in order to prevent or mitigate CRA. It is apparent that there is not yet a clear terminology in place when describing atrophic changes in VN-treated IRD. There is no Medical Dictionary for Regulatory Activities preferred term for CRA, although a Human Phenotype Ontology term exists for CRA (identifier HP:0000533), which is defined as atrophy of the choroid and retinal layers of the fundus and is stated to be synonymous with chorioretinal thinning and choroidal sclerosis. In the Luxturna summary of product characteristics, CRA is an umbrella term that may include adverse events such as retinal degeneration, retinal depigmentation, and injection site atrophy.^12^ In the literature, terms including atrophy, retinal atrophy, CRA, and RPE atrophy are all used to describe atrophic changes, sometimes interchangeably within the same publication. In some cases, CRA at the injection site is reported separately from CRA elsewhere, while in other cases, there is no distinction made between these presentations of CRA. Chorioretinal atrophy is generally described both in terms of its relation to the injection site or bleb, and by nearby anatomical features.

This lack of consistency could hinder the communication of research findings, slowing progress in understanding this phenomenon. Standard terminology and metrics for CRA, agreed upon by the scientific community, would support future research efforts by providing greater consistency across different studies and facilitating comparison and integration of results between research groups. Here we propose a naming convention for CRA, as well as suggest some metrics and other information that future studies could collect as standard.

### CRA Classification

A previous publication has proposed a classification of CRA according to a qualitative judgment of the pattern of atrophy, dividing CRA into atrophy at the injection site (touchdown atrophy), coin-shaped (or nummular) areas of atrophy predominantly involving the periphery (nummular atrophy), and atrophy predominantly in the perifoveal region (perifoveal atrophy).^26^ Although this proposed classification is a valuable starting point for categorizing CRA, we feel that a naming strategy more closely linked to both treatment and location may be more clinically relevant for studies aiming to provide insights into CRA after treatment with VN.

**Figure 1:**
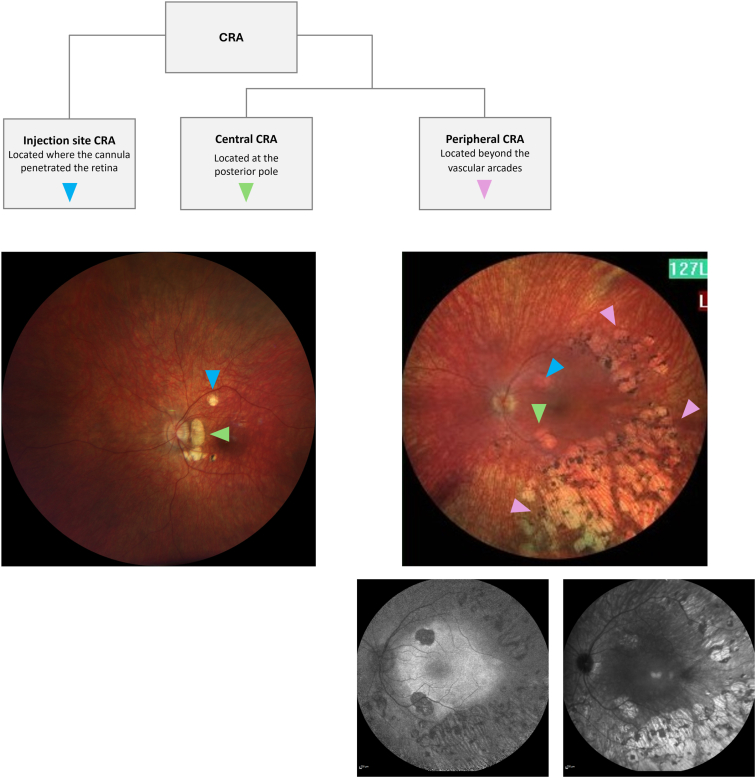
Proposed terminology for definition of CRA. Example image for injection site CRA and central CRA represents the left eye of an 8-year-old female, 2 years after VN treatment; injection site CRA can be observed just beneath the superior temporal vascular arcade, whereas central CRA in the treatment area is present in the area temporal to the optic disc. On OCT, subretinal deposits, likely representing inflammatory cells, were noticed in that region at 4 to 6 weeks after VN treatment. Image provided by Bart P. Leroy. Example image for peripheral CRA shows a typical CRA fundus picture with the corresponding fundus autofluorescence (bottom left) and IR (bottom right) images. Image provided by Katarina Stingl. CRA = chorioretinal atrophy; IR = infrared reflectance; VN = voretigene neparvovec.

We propose using the term “injection site CRA” to refer to CRA occurring specifically at the location where the cannula penetrated the retina. For all other CRA, we propose defining the atrophy based on its retinal localization, as “central CRA” and/or “peripheral CRA” (Fig 1). Central CRA would be defined as CRA occurring at the posterior pole within the VN treatment area. It is often perifoveal as it frequently seems to spare the fovea. Chorioretinal atrophy occurring in this region might therefore be expected to be treatment-related, with the caveat that it is not possible to determine the precise position of the bleb area, as subretinal fluid can shift after the conclusion of surgery. While the location of the bleb may be clearly apparent immediately after injection, factors such as noncompliance with supine positioning may cause the bleb to shift,^15^ and the use of air tamponade has been shown to result in a wide subretinal diffusion of the virus vector away from the injection site and beyond the surgical bleb borders.^52^ Peripheral CRA would be defined as CRA occurring beyond the vascular arcades. This form of CRA might be more likely to reflect the natural course of disease progression but to date may have been underreported, since follow-up in VN-treated patients typically focuses on the posterior pole. A dedicated term for this form of CRA could encourage increased documentation and potentially aid in confirming its etiology or relationships.

### Monitoring Patients Treated with VN

Combining imaging modalities such as OCT, FAF using both blue (short wavelength) and near-infrared (short wavelength) light, near-infrared reflectance, and widefield fundus photography provides a comprehensive view of the outer retina in *RPE65*-IRD. Regular follow-up visits with these methods are recommended in patients treated with VN, to track disease evolution and treatment effects, including CRA.^20^ At a minimum, OCT and FAF may be used, complemented with other modalities if available. When treating a patient with VN, we recommend that a record is made of the size and location of the bleb at the time of the procedure (ideally using intraoperative OCT), as well as the location of the retinotomy and other details relating to the procedure that could potentially affect the distribution of vector (e.g., the use of air exchange or air tamponade).

Close monitoring for signs of inflammation after treatment, in both the vitreous and subretinal spaces, is recommended, particularly once standard prophylactic immunosuppressant therapy has been completed.^16^ Any inflammation should be treated appropriately. Follow-up of patients treated with VN should continue for as long as possible, ideally for the life of the patient. A suggested workflow of examinations for evaluation of safety and efficacy in patients receiving VN is provided in Table 2.

**Table 2:** Recommended Monitoring Schedule for Patients Receiving Voretigene Neparvovec

	Before Treatment	Months after Treatment				Annually
		0.5	1	3	6	
Patient history		+	+	+	+	+
BCVA and slit lamp	+	+	+	+	+	+
FST or DAP where available and appropriate∗	+		+			+
OCT	+	+	+	+	+	+
(Ultra) wide-angle photography	+	+	+	+	+	+
FAF imaging (blue and near-infrared light)	+	+	+	+	+	+
NIR imaging	+	+	+	+	+	+

BCVA = best-corrected visual acuity; DAP = dark-adapted perimetry; FAF = fundus autofluorescence; FST = full-field stimulus threshold; NIR = near-infrared reflectance.

∗ FST to include testing using blue and red light, if possible. FST and DAP are not recommended for younger children.

Fundus autofluorescence imaging, especially 55-degree blue-light FAF, is useful for detecting and delineating CRA in *RPE65*-IRD patients, even though the FAF signal is typically extremely weak in these cases. In such circumstances, near infrared reflectance imaging may be superior. However, improved FAF signals may be observed posttreatment, aiding in early atrophy detection.^18^^,^^20^ Although short-wavelength light can be phototoxic at sufficiently high intensities or durations, there is currently no evidence that the brief exposures used in clinical imaging cause retinal damage. Blue-light FAF is widely used in routine practice, and its diagnostic value for identifying CRA is well-established. Clinicians may nonetheless wish to be mindful of potential light-exposure considerations, particularly in the early posttreatment period, and balance these against the valuable contribution that blue-light FAF can make to CRA assessment.

OCT is recommended for diagnosis and staging of atrophy, as well as for the evaluation of postoperative preretinal and subretinal inflammation. However, as many patients treated with VN have poor fixation, this can result in suboptimal quality OCT imaging.^26^ We nevertheless recommend maximal efforts to obtain interpretable OCT images.

For patients in whom CRA is observed after treatment, we suggest documenting the classification of CRA according to the terminology proposed earlier, as well as performing detailed retinal imaging to provide a visual record of the number, size, appearance, and location of the atrophic lesions. If possible, rod-related functional outcomes should be measured using tools such as FST and dark-adapted perimetry. Based on reported correlations with initial posttreatment functional rescue, these techniques may help to better estimate the patient risk profile.^17^ Standard kinetic perimetry may also be useful to monitor for any long-term effects of CRA on peripheral vision under standard conditions, and has the benefits of being easier to perform and more widely available than dark-adapted perimetry.

In the future, it is possible that artificial intelligence may play an increasing role in CRA monitoring, providing automated detection and quantification of CRA area to better and more precisely monitor progression. In the meantime, it is our hope that systematically recording cases of CRA in the manner proposed will help to provide valuable information that may be compared and collated across centers, providing insights into the causes, outcomes, and possibly the prevention or avoidance of some cases of post-VN CRA. Due to the rarity of *RPE65*-associated IRD, this may take considerable time to achieve. Until then, patients eligible for VN therapy should be informed before consenting to treatment about the current knowledge and known predispositions for the development of treatment-linked CRA. Any discussion of risk should be balanced by the reassurance that, when CRA does occur, its impact on functional rescue is typically limited.

## Conclusions

Significant progress has been made in characterizing and understanding the etiology of CRA after VN treatment, with an increasing number of documented cases contributing to a growing body of literature. However, numerous questions remain, which will require further clinical experience and research to answer. In this publication we have discussed hypotheses on CRA etiology, proposed a standard terminology and key metrics for monitoring CRA to support future research efforts, and suggested some measures that clinicians treating RPE65 patients with VN can consider implementing in the meantime, including postprocedure monitoring, and follow-up for patients who develop CRA.

## Data Availability

The data for this article consist of bibliographic references, which are included in the References section.
